# Study protocol: home-based telehealth stroke care: a randomized trial for veterans

**DOI:** 10.1186/1745-6215-11-74

**Published:** 2010-06-30

**Authors:** Neale R Chumbler, Dorian K Rose, Patricia Griffiths, Patricia Quigley, Nancy McGee-Hernandez, Katherine A Carlson, Phyllis Vandenberg, Miriam C Morey, Jon Sanford, Helen Hoenig

**Affiliations:** 1 Department of Veterans Affairs Health Services Research & Development (HSR&D) Center of Excellence on Implementing Evidence-Based Practice (CIEBP), Richard L. Roudebush VA Medical Center, Indianapolis, Indiana, USA; 2 VA HSR&D Stroke Quality Enhancement Research Initiative (QUERI) Program, Richard L. Roudebush VAMC, Indianapolis, IN, USA; 3 Department of Sociology, Indiana University School of Liberal Arts, Indiana University Purdue University Indianapolis, Indianapolis, IN, USA; 4 Regenstrief Institute, Indianapolis, IN, USA; 5 Department of Physical Therapy, College of Public Health and Health Professions, University of Florida, Gainesville, FL, USA; 6 Rehabilitation Research & Development (RR&D) Brain Rehabilitation Research Center, North Florida/South Georgia Veterans Health System, Gainesville, FL, USA; 7 Atlanta VA Rehab R&D Center, Atlanta VAMC, Decatur, GA, USA; 8 Division of Geriatrics and Gerontology, Emory University School of Medicine, Atlanta, GA, USA; 9 HSR&D/RR&D Research Center of Excellence: Maximizing Rehabilitation Outcomes, James A. Haley VAMC, Tampa, FL, USA; 10 Physical Medicine & Rehabilitation Service, Durham VAMC, Durham, NC, USA; 11 Geriatric Research Education and Clinical Center, Durham VAMC, Durham, NC, USA; 12 Department of Medicine, Duke University Medical Center, Durham, NC, USA; 13 Center for the Study of Aging and Human Development and Older Americans Independence Center, Duke University Medical Center, Durham, NC, USA; 14 College of Architecture, Georgia Institute of Technology, Atlanta, GA, USA

## Abstract

**Background:**

Stroke is one of the most disabling and costly impairments of adulthood in the United States. Stroke patients clearly benefit from intensive inpatient care, but due to the high cost, there is considerable interest in implementing interventions to reduce hospital lengths of stay. Early discharge rehabilitation programs require coordinated, well-organized home-based rehabilitation, yet lack of sufficient information about the home setting impedes successful rehabilitation. This trial examines a multifaceted telerehabilitation (TR) intervention that uses telehealth technology to simultaneously evaluate the home environment, assess the patient's mobility skills, initiate rehabilitative treatment, prescribe exercises tailored for stroke patients and provide periodic goal oriented reassessment, feedback and encouragement.

**Methods:**

We describe an ongoing Phase II, 2-arm, 3-site randomized controlled trial (RCT) that determines primarily the effect of TR on physical function and secondarily the effect on disability, falls-related self-efficacy, and patient satisfaction. Fifty participants with a diagnosis of ischemic or hemorrhagic stroke will be randomly assigned to one of two groups: (a) TR; or (b) Usual Care. The TR intervention uses a combination of three videotaped visits and five telephone calls, an in-home messaging device, and additional telephonic contact as needed over a 3-month study period, to provide a progressive rehabilitative intervention with a treatment goal of safe functional mobility of the individual within an accessible home environment. Dependent variables will be measured at baseline, 3-, and 6-months and analyzed with a linear mixed-effects model across all time points.

**Discussion:**

For patients recovering from stroke, the use of TR to provide home assessments and follow-up training in prescribed equipment has the potential to effectively supplement existing home health services, assist transition to home and increase efficiency. This may be particularly relevant when patients live in remote locations, as is the case for many veterans.

**Trial Registration:**

Clinical Trials.gov Identifier: NCT00384748

## Background

Stroke patients clearly benefit from intensive and coordinated inpatient care. Access to post-acute stroke in-patient rehabilitation is limited for many individuals, especially those residing in rural locations. One study found that over 75% of US veterans treated within the Department of Veterans Health Administration (VHA) with an acute stroke are cared for in hospitals without an inpatient rehabilitation bed-unit (RBU), and fewer than 10% of stroke patients are transferred to a RBU from facilities without a RBU [1].

While inpatient rehabilitation care is the preferred form for many patients post-stroke, due to access and financial barriers, many patients do not have this option. Another viable option is home-based rehabilitation. The ultimate goal of home-based rehabilitation is for stroke patients to obtain optimal functioning at home. Unfortunately, resources for in-home rehabilitation are limited [2]. In the rehabilitation field, "telerehabilitation" (TR; use of home-telehealth technologies to provide distance support, rehabilitation services and information exchange between people with disabilities and their clinical providers) represents an approach for potentially addressing this access to post-acute rehabilitation care problem for stroke patients [3].

TR is particularly well-suited to address the rehabilitation needs for those with stroke in several ways. First, TR offers increased access to post-acute rehabilitation following hospitalization. Second, TR could be used in consultation with providers who treated the patient in the hospital, improving coordination of care. Third, TR could reduce the need to travel between patients' homes and the rehabilitation clinic, a particular benefit for those in rural areas because they face incomplete service networks that threaten their safety and independent functioning [4]. This is particularly relevant to VHA where approximately 40% of its 8 million enrollees live in rural areas [5]. Fourth, TR could be applicable for home health nurses to use and provide therapists with important information prior to a home visit, increasing efficiency and effectiveness. TR takes advantage of novel technology to deliver a focused rehabilitation intervention that complements currently available post-acute rehabilitation resources.

Our on-going intervention, the home-based Stroke Tele-Rehabilitation (STeleR) trial, extends interventions used in previous research in several ways. Therapy is delivered by occupational and physical therapists to stroke patients via TR strategies, an activity that has been rarely done, but has been recommended recently in a Scientific Statement from the American Heart Association/American Stroke Association [3]. Also, contrary to most previous TR studies, our study employs a multifaceted intervention that simultaneously evaluates the home environment, assesses the patient's functional mobility skills, initiates rehabilitative treatment, prescribes exercises tailored for stroke patients and provides periodic goal-oriented reassessment, feedback and encouragement. This paper describes the aims and objectives, study design, recruitment strategy and analytic procedures of this ongoing prospective, innovative STeleR RCT.

### Study Aims and Objectives

The objective of the study is to examine a TR intervention that uses telehealth technology to improve outcomes of patients post-stroke after discharge home. The primary aim is to determine the effect of TR on physical function. Secondary aims are to determine the effect of TR on: disability; falls related self-efficacy (confident of not falling); and patient satisfaction (satisfaction with health care services received since the stroke).

The primary research question and the four secondary research questions are:

#### Primary Research Question

Over the 6-month study period, does the TR group have greater improvement in physical function (motor subscale on the telephone version of the Functional Independence Measure [FONEFIM] [6,7] than a Usual Care group?

#### Secondary Research Question 1

Over the 6-month study period, does the TR group have greater improvement in disability (Disability component of the Late Life Function and Disability Instrument) [8] than a Usual Care group?

#### Secondary Research Question 2

Over the 6-month study period, does the TR group have greater improvement in falls-related self-efficacy (the Falls Self Efficacy Scale) [9] than a Usual Care group?

#### Secondary Research Question 3

Over the 6-month study period, does the TR group have greater improvement in patient satisfaction (the Stroke Specific Patient Satisfaction with Care Scale) [10] than a Usual Care group?

#### Secondary Research Question 4

Over the 6-month study period, do patients, regardless of intervention arm, discharged from Veterans Affairs Medical Centers (VAMC) with a RBU have greater improvements in function (motor sub-scale of the FONEFIM) than those from VAMCs without a RBU?

## Methods

### Setting and Overall Study Design

**Figure 1:**
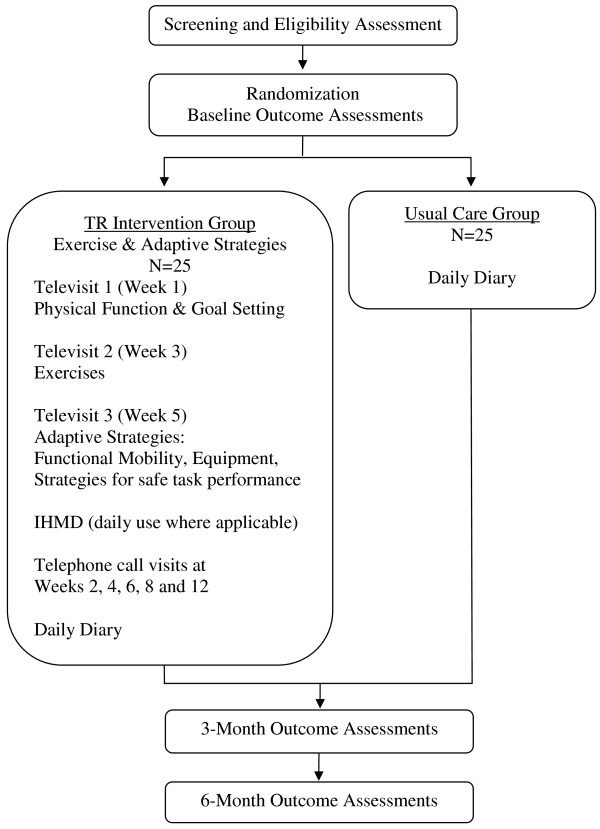
**Design and Study Flow Chart**.

This is a three-site, 2-arm, single-blinded RCT with enrollment at three VAMCs: 1) James A. Haley VAMC in Tampa, FL; 2) Atlanta VAMC, Decatur, GA; and 3) Durham VAMC, Durham, NC. The James A. Haley VAMC facility has a RBU, while the other two VAMCs do not have a RBU. However, the three facilities are otherwise fairly similar. The Richard L. Roudebush VAMC, Indianapolis, IN is the Coordinating Center. Eligible patients are randomized into the TR intervention or Usual Care groups. During the study, all participants receive usual care from their providers. The TR intervention is delivered over 3 months via home visits and telephone calls. Outcome assessments are conducted at baseline, 3-months and 6-months. Figure 1 outlines the study design.

### Ethics

The Roudebush VAMC (Indianapolis, IN) received approval for this study from the IUPUI/Clarian Institutional Review Board (#0804-54) and the Roudebush VAMC Research and Development (R&D) Committee. The enrolling sites each received approval from their respective Institutional Review Boards and/or VAMC R&D Committees. All study participants gave written consent prior to participation.

### Patient Population and Recruitment

The study population consists of veterans who have experienced an ischemic or hemorrhagic stroke within the preceding 24 months. For the purposes of our study, stroke is defined as "a rapid onset event of vascular origin reflecting a focal disturbance of cerebral function, excluding isolated impairments of higher function and persisting longer than 24 hours" [11] per the criteria of Reker et al. [12]. Other inclusion criteria include age between 45-90; discharged to the community; Short Portable Mental Status Questionnaire (SPMSQ) with no more than 4 errors to ensure cognitive intactness; discharge motor FIM score of 17-88 (i.e., maximal assistance on no more than 4 motor activities of daily living (ADLs) as the most severe stroke included and modified independence on at least 2 motor ADLs as the least severe); able to follow a 3-step command; concurrence by the patient's physician; signed VHA Medical Media release form; and signed informed consent.

We use three complementary methods to identify potential participants via a three-step recruitment process. The first method utilizes the VA Administrative data source, the automated Functional Status Outcomes Database (FSOD) notification system, which uses admission diagnoses to notify clinicians that a patient with acute stroke has been admitted to the hospital. Potential study participants are also identified by referrals made to study staff by clinicians on the acute medical/surgical wards, and stroke or rehabilitation units. In a third method, site specific data bases are queried by analysts employing an algorithm similar to the FSOD automated search strategy. Potential participant lists are reviewed by study staff for determination of study eligibility. Randomization to group occurs after final approval by the patient's primary care provider (PCP) and discharge to the community.

### Randomization

Study participants are randomly assigned to one of two groups: TR intervention or Usual Care. Previous studies found differences in process and outcomes across VAMCs with and without RBUs [1]. Therefore, the presence or absence of a RBU is treated as a factor in the design and participants are randomly assigned to TR or to Usual Care within each site. So as not to unduly constrain recruitment within the individual sites, the one RBU site (Tampa VAMC) and the two non-RBU sites (Durham and Atlanta VAMCs) will be allowed to vary in the number of recruited patients. Randomization is performed by centrally-sealed allocation.

Treatment allocation occurs when a study participant meets the inclusion criteria, and signs the HIPAA and informed consent forms. The teletherapist registers the participant into the database, which in turn asks if the participant is ready to be randomized. After the teletherapist enters yes, the site-specific randomization program behind the form displays the participant's group assignment number (treatment versus control). Study participants are randomized in blocks of random sizes of 2 and 4 at each site. Site-specific randomization lists were computer-generated (i.e., generated by an individualized basic visual code program) and were concealed from the researchers by a senior data manager who is not involved in the study. This information remains confidential and is not shared with the study sites in concordance with the CONSORT guidelines. This trial uses a prospective, randomized, outcome-blinded design, in which all outcome assessments are assessed by a research assistant blinded to treatment allocation and uninvolved in consenting and management of the patients.

### TR Intervention

#### Overview

The TR intervention consists of two components that target safe functional mobility of the individual within an accessible home environment: 1) exercise and 2) adaptive strategies. The TR intervention takes place over three months (Figure 1). This 3-month intervention consists of the following three components: 1) three one-hour home visits (hereafter referred to as "televisits") by a trained assistant to assess physical performance and help communicate interventions recommended by the teletherapist; 2) daily participant use of an in-home messaging device that is monitored weekly by the teletherapist, for those participants with an active analog telephone line; and 3) five telephone intervention calls between the teletherapist and the participant. Over this three month period, participants are able to receive routine VA care as directed by their physicians, thus the intervention serves as a supplement to existing home health care for stroke survivors rather than a substitute.

The three televisits occur within five weeks post randomization (Weeks 1, 3, and 5), approximately one visit every 12-16 days. The five telephone call "visits" occur during weeks 2, 4, 6, 8 and 12. One additional televisit may be scheduled as needed at the teletherapist's discretion. This schedule allows flexibility to plan study visits around other prescribed home health visits, medical services and the participant's daily activities. Each televisit is videotaped and focuses on a particular aspect of the intervention. The participant's privacy is protected at all times during every visit by not filming their face or referring to them by their full name. The televisit intervention is based in large part on the TR intervention tested in our previous study with the addition of 5 telephone call visits to allow the TR treatment program to be advanced as the participant progresses [13,14].

#### Televisits

##### Televisit 1

The first televisit is devoted to assessment of physical function and goal-setting. During this first visit, the assistant installs and instructs the participant in the use of the in-home messaging device (IHMD) if the participant has an analog home telephone line (see section '*In Home Messaging Device*'). The assistant uses a camcorder to record the hone environment and the participant carrying out standardized measures of physical and functional performance, as described below.

The assessment of physical function includes measures of static and dynamic sitting, standing balance and upper extremity range of motion. The battery includes the Performance-Oriented Motor Assessment-Balance (POMA-B) scale [15], a widely used tool for assessing mobility and falls risk in older people. Functional active range of motion of the upper extremities is measured using a protocol developed by Jette and Branch [16], and includes shoulder abduction/external rotation, mass finger flexion and extension. Additionally, functional mobility is assessed as the participant demonstrates toilet and tub/shower transfers. Participants are instructed to perform these tasks in their usual and customary method. Finally, the participant demonstrates any previously prescribed or voluntary exercises that he or she may already be doing to allow the teletherapist to assess the quality of their performance and to avoid any redundancy in the therapeutic regimen.

Collectively, the assessments inform the selection of targeted exercises and adaptive strategies for each participant. The teletherapist carefully reviews the videotape after the home visit. All treatment/equipment recommendations are incorporated into a written "Adaptive Prescription".

#### Exercise Intervention

Regaining the ability to walk is the number one stated goal for individuals post-stroke [17]. Therefore, the exercise intervention is functionally-based, and developed from successful trials for mobility disability in the geriatric and stroke population [18-21]. The exercise portion of the Adaptive Prescription includes 3-4 exercises that focus on strength and balance. There are two different sets of exercises from which the teletherapist makes selections: one set has exercises for participants who are ambulatory/able to stand independently, while the other has exercises for participants who are non-ambulatory/unable to stand independently. Table 1 presents detailed descriptions of each exercise by set. Each participant receives their exercise prescription when the Adaptive Prescription is delivered during televisit two.

**Table 1:** Exercises

A. Exercises for ambulatory/able to stand independently participants
*Hip and Knee Bends*

1. While standing facing kitchen sink (choose one):
a. Hold with both hands
b. One hand
c. Fingertips
2. Bend hips and knees
3. Rise up again

*Heel Raises*

1. While standing facing kitchen sink (choose one):
a. Hold with both hands
b. One hand
c. Fingertips
2. Rise up on your toes
3. Hold for count of 5
4. Come down
Progression:
A. Standing on both legs, hold with both hands.
B. Standing on both legs, hold with one hand.
C. Standing on both legs, hold with fingertips.
D. Standing on one leg, hold with both hands.
E. Standing on one leg, hold with one hand.
F. Standing on one leg, hold with fingertips.

*Sit to Stand*

This activity involves standing up from a seated position and returning to a seated position with weight evenly distributed on both legs.
Progression:
A. Sitting at edge of chair, lean forward, push up with arms and stand up.
B. Sitting at edge of chair, lean forward, put arms out and stand up.
C. Sitting at edge of chair, lean forward, fold arms across chest and stand up.
D. Sitting at back of chair, lean forward, push up with arms and stand up.
E. Sitting at back of chair, lean forward, put arms out and stand up.
F. Sitting at back of chair, lean forward, fold arms across chest and stand up.

*Marching in Place*

This activity involves standing on a level surface and raising knees, in an alternating manner, as high as possible.
Progression:
A. Holding on with both hands, raise legs, in an alternating manner, bending hip to 90 degrees.
B. Holding on with one hand, raise legs, in an alternating manner, bending hip to 90 degrees.
C. Without holding on, raise legs, in an alternating manner, bending hip to 90 degrees.

*Tandem Walking*

1. Stand with (right/left) hand on counter.
2. Move hand along counter as you step.
3. Place right heel directly in front of toes of left foot.
4. Now place left heel directly in front of toes of right foot.
5. Continue until you run out of counter space.
6. Walk backwards, placing toe directly behind heel for each step.
7. Continue until you are back to your starting position.

*Dynamic Standing Balance*

1. Turning towards (right/left) side
2. Turning towards (left/right) side

**B. Exercises for non-ambulatory/unable to stand independently participants**

*Bridging*

1. Bend knees and place hands at sides.
2. Raise buttocks off surface of the bed and hold for 3 seconds, resume starting position.
Progression:
A. Bilateral bridge.
B. Bilateral bridge: shift hips from side to side, return to start position
C. Unilateral bridge: one leg bent with the other leg straight

*Leg Crossover*

1. Start with leg flat on floor and other leg bent.
2. Lift bent leg over other leg.
3. Lift and uncross, resuming position in #1.
4. Repeat crossing/uncrossing motion on both sides.

*Heel Slides*

1. Start with knees bent, feet resting on floor.
2. Slowly slide heel of one leg down and straighten leg.
3. Slowly bring heel of leg along floor, and return to start position (keep heel in contact with floor throughout exercise).
4. Start with #1 and repeat with other leg.

*Side-lying to Sit*

While lying on (left/right) side with hips and knees bent and heels supported by the bed:
1. Push down with the elbow touching the surface of the bed and the opposite hand.
2. Come to a sitting position with legs dangling off edge of bed.

*Static Sitting Balance*

Progression:
A. Sitting with equal weight on both buttocks for 30 seconds.
B. Sitting with upright posture:
1. Shift weight to hip, lift opposite leg from chair by flexing hip. Hold 5 seconds.
2. Shift weight to opposite hip, lift leg from chair by flexing hip. Hold for 5 seconds.

*Dynamic Sitting Balance*

1. Same side, lean forward in chair, diagonal reaching with stronger arm.
2. Same side, backward diagonal reaching with stronger arm.
3. Same side, lean forward, diagonal reaching with weaker arm.
4. Same side, backward diagonal reaching with weaker arm.

##### Televisit 2

The exercise component of the TR intervention is the primary focus of televisit 2. By this time, the teletherapist has met with the assistant to discuss the participant's prescription and provides protocols for the instruction and safe execution of each exercise. The assistant brings the exercise prescription and pertinent exercise handouts for the participant to the second televisit. The assistant explains and demonstrates each exercise before videotaping the participant performing each exercise for later review by the teletherapist. Upon arrival to the participant's home, the assistant checks the participant's blood pressure and heart rate to determine that the values are within the pre-established ranges. If a value is outside its pre-established range, the participant is not asked to participate in any exercises during this televisit. If new equipment was ordered from televisit 1, and has been delivered, instructions on how to use the new equipment may be presented to the participant as well during televisit 2 (time permitting). Generally, training in use of adaptive methods and adaptive equipment for bathroom mobility takes place during televisit 3.

##### Televisit 3

Televisit 3 primarily focuses on functional mobility using the adaptive strategy component of the TR intervention. The functional training component of the Adaptive Prescription(see example below) uses an "environmental approach" to resolve functional problems (i.e., change the environment or change the interaction with the environment), and includes use of assistive technology, environmental modifications, adaptive strategies, and education, based on prior work by Hoenig et al [13]. The functional component of the Adaptive Prescription is developed through dialogue and discussion with the participant, after the teletherapist has reviewed the initial videotape of the participant's performance and the assistant in the home. For the identified performance areas, the teletherapist probes about (a) the specific nature of the problem, (b) how a typical day proceeds (e.g., *"Tell me about a typical day in which this problem occurs ..."*), (c) current strategies in place to handle the identified problem (e.g., *"Tell me how you handle this ..."*), and (d) relevant interactions with caregivers. The teletherapist conducts an in-depth examination with the participant and assistant of the positive and negative factors that influence this particular performance area, and jointly specify goals and methods to address the performance problem. The assistant reviews the adaptive prescription with the participant. If the teletherapist recommends new adaptive techniques, the assistant reviews these with the participant, providing the participant with relevant handouts developed for the study, demonstrating the novel methods and videotaping the participant's performance with the functional mobility tasks and the relevant equipment. If adaptive equipment or assistive technology has been delivered to the home, the assistant verifies it has been installed correctly and reviews its proper use with the participant per the detailed study protocols. The participant's exercises are reviewed and exercise performance is also videotaped during the third televisit.

##### Example of adaptive prescription toileting (functional component)

1. Equipment

a) Electronic controls (e.g., clapper, motion detector)

b) Oxygen tank w/extra tubing/portable tank

c) Mobility Aid: walker, cane, crutch, wheelchair

d) Lift: Mobile (e.g. Hydraulic/Mechanical), Overhead

e) Gait belt

f) Sliding, transfer board

g) Bedside Commode

h) Urinal

i) Catheter with bag

j) Other: ___________________

2. Physical/spatial modifications

k) Change floor surface (replace floor, remove rugs or obstacles)

l) Add lighting (e.g., new fixtures, change curtains, night light)

m) Decrease glare

n) Change light controls (move switch)

o) Remove unnecessary objects: throw rug, shower curtain, towels, etc

p) Rearrange furniture or bathroom fixtures to create more space

q) Remove walls (e.g. closet) or add on to bathroom to create more space

r) Use a different toilet: commode chair, use another bathroom, add a new bathroom

s) Install new grab bars

t) Replace or move existing grab bars

u) Change toilet height: replace toilet, raised toilet seat, donut, safety frame, toilevator

v) Stabilize toilet

w) Other: ___________________

3. Adaptive methods

x) Use a urinal or different bathroom

y) Bed mobility method

z) Bed mobility training and practice

aa) Transfer method

bb) Transfer training and practice

cc) Stay within "triangle of efficiency"

dd) Catheterization

ee) Bowel program

ff) Prompted voiding

gg) Use diapers

hh) Other: ___________________

4. Energy conservation

ii) Take your time

jj) Go to the toilet more often, so you don't have to rush

kk) Use a nearby toilet: commode, different bathroom

ll) Prepare ahead (e.g., toilet paper near, other items for toileting, etc.)

mm) Adjust equipment before changing position

nn) Avoid transfers when tired

oo) Ask for help if you feel sick or especially tired

pp) Allow time for eyes to adjust to change in lighting

At the discretion of the teletherapist, a prn visit (televisit 4) is performed in the event that interval problems arise that cannot be addressed telephonically and that do not require medical evaluation at the hospital (e.g., a minor fall and the circumstances of which cannot be adequately addressed telephonically).

#### Telephone Intervention

There are a total of five telephone calls from the teletherapist to the participant. The first telephone call occurs during Week 2 (approximately 7-10 days after televisit 1). During the first call, the teletherapist establishes rapport with the participant by commending them on what they did appropriately and providing positive feedback on their performance as appropriate. The call allows for clarification of any concerns the teletherapist may have, reviewing the participant's current exercise program, discussing participant preferences and soliciting cooperation and input from the participant. Some preliminary treatment or exercise recommendations may be made at this time, if appropriate, and any potentially dangerous activities viewed on the videotape are discussed in detail with alternative strategies provided. If the videotaped evaluation shows that assistive technology or adaptive equipment is needed, the teletherapist asks the participant for permission to order relevant equipment through the VAMC.

If the IHMD was not set up in the home due to lack of an analog telephone line, the teletherapist uses the exercise dialogue from the IHMD to query the participant about exercise adherence. The teletherapist discusses impediments to participants performing exercises and engages the participant in troubleshooting ways to get around barriers in order to encourage adherence to the exercise program. The exercise dialogue enables flexibility such that participant needs drive the content of the call. This exercise dialogue was derived from recently published clinical trials that were used to promote improved physical activity [22,23]. Table 2 presents the contents of the 'Exercise Adherence Dialogue Response Form'.

**Table 2:** Exercise adherence dialogue response form

	Teletherapist questions:	Responses:		
1.	In the last **WEEK**, has anything happened to your **PHYSICAL **health or in your life that we should know about that will prevent you from starting your exercise routine?	**Yes**	**No**	

2.	If response is **Yes**: "I am sorry to hear that. If you feel dizzy, have problems with your speech, eyesight, or increased weakness, you should call your doctor or 911."			

3.	Let's take a look at the exercise plan that we put together. Have you been able to follow this plan?	**Yes**	**No**	

4.	If answer is **Yes**, "That is great! Keep up the good work, and continue to exercise as often as you are able to do so."			

5.	If answer is **No**, go to question #28.			

6.	So, let us take a look at your **BALANCE ACTIVITIES**. In the past week, did you do the exercises we gave you?	**Yes**	**No**	**N/A**

7.	Exactly how many **TIMES **last week were you able to do the balance activity?	**Times**		

8.	Exactly how many **MINUTES **were you able to do the balance activity each time?	**Minutes**		

9.	So, let us take a look at your **STRENGTH ACTIVITES**. In the past week, did you do the exercises we gave you?	**Yes**	**No**	**N/A**

10.	Exactly how many **TIMES **last week were you able to do the strength activity?	**Times**		

11.	Exactly how many **MINUTES **were you able to do the balance activity each time?	**Minutes**		

12.	Please tell me what **EXERCISES **you have been able to do this week.			

	**Exercises for non-ambulatory/unable to stand independently:**			

13.	Did you do the **BRIDGING **exercise?	**Yes**	**No**	**N/A**

14.	Did you do the **LEG CROSSOVER **exercise?	**Yes**	**No**	**N/A**

15.	Did you do the **HEEL SLIDES **exercise?	**Yes**	**No**	**N/A**

16.	Did you do the **SIDE-LYING TO SIT **exercise?	**Yes**	**No**	**N/A**

17.	Did you do the **SITTING BALANCE **exercise?	**Yes**	**No**	**N/A**

18.	Did you do the **DYNAMIC SITTING BALANCE **exercise?	**Yes**	**No**	**N/A**

19.	OK.			

20.	**Exercises for ambulatory/able to stand independently:**			

21.	Did you do the **HIP AND KNEE BENDS **exercise?	**Yes**	**No**	**N/A**

22.	Did you do the **HEEL RAISES **exercise?	**Yes**	**No**	**N/A**

23.	Did you do the **SIT TO STAND **exercise?	**Yes**	**No**	**N/A**

24.	Did you do the **MARCHING IN PLACE **exercise?	**Yes**	**No**	**N/A**

25.	Did you do the **TANDEM WALKING **exercise?	**Yes**	**No**	**N/A**

26.	Did you do the **DYNAMIC STANDING BALANCE **exercise?	**Yes**	**No**	**N/A**

27.	OK.			

28.	What is preventing you from doing your **PRESCRIBED PHYSICAL ACTIVITIES?**	1. Not enough time to exercise		

		2. Not enough strength or energy		

		3. Do not understand how to do the exercise(s)		

		4. I need some help with the exercise(s)		

		5. Other:		

29.	What would make it easier for you to be active on a regular basis?	1. To make more time		

		2. Ask someone to explain how to do exercise(s)		

		3. Ask for help with exercise(s)		

		4. Other:		

30.	Do you still have trouble with balance activities?	**Yes**	**No**	**N/A**

31.	Do you still have trouble with strength activities?	**Yes**	**No**	**N/A**

32.	This is important for your recovery, well-being and overall health. Keep going.			

33.	Please review the set exercises that we have given you. These exercises will show you how to build back your strength.			

34.	Will you agree to do this?	**Yes**	**No**	**N/A**

35.	If answer is **NO**, "I'm sorry to hear that, but don't give up, keep trying during the next week."			

Telephone call visits 2-5 at Weeks 4, 6, 8 and 12 are directed towards reassessment and progression of the exercise program and the adaptive strategies, along with reviewing participant concerns pertaining to the toilet and tub/shower transfers and/or exercise adherence. The study protocol is designed to allow some flexibility in both the home visits with the assistant and the telephone calls with the teletherapist to enable the participant's most immediate needs to be addressed and to accommodate obtaining recommended adaptive equipment.

### Usual Care Group and Other Medical Care

Study participants randomized to Usual Care are informed that they will not get any extra care as part of the study. There are no attempts by study personnel to influence their self-care activities or physical functioning. The study's design ensures that both the TR and the Usual Care groups get any equivalent home health services that are provided as part of routine VA care, such as home health care by a nurse or nurse's aide, and/or visits by rehabilitation practitioners.

#### Telecommunication Technology

The study tried using several forms of technology to facilitate live two-way televisits between the teletherapist at the hospital and the participant and assistant in the home, including use of an analog videophone linked to a wireless transmitter and videocamera, and a wireless broadband internet system using a streaming Universal Serial Bus (USB) webcam linked to a VA encrypted laptop and virtual private network (VPN) to securely access the internet. However, the functionality of both systems for live two-way communication and capture of gross and fine motor function was erratic, so our protocol primarily relies on the videotaped recording of the session. The videocamera recording is very high quality and enables the teletherapist to assess fine motor movement and environmental characteristics. Depending on the assessment, the videocamera is either placed on an appropriately located tripod or hand carried by the assistant as the participant progresses through specific functional tasks. The assistants and teletherapists were all trained jointly in use of the technology, supplemented by detailed protocols already developed. Further, a training videotape was developed for new employees to view and for staff to use as a reference point of information.

#### In Home Messaging Device

The purpose of the IHMD is two-fold: to screen for unforeseen problems (i.e., allow for more efficient use of teletherapist time during the TR intervention) by identifying, a priori, potential issues or difficulties the study participant may be experiencing with recommended treatment and, to reinforce adherence to the recommended exercises. Due to its operability, the IHMD is employed only with those TR intervention participants who have an active analog telephone line in the home. The teletherapist views all the participant's answers via the Internet through software called the iCare Desktop. The IHMD uses standardized and validated scales targeting common post-stroke complications (depression, self-care/mobility, and falls) that might affect the success of the TR intervention or might be effectively treated by the TR.

Depression is common after stroke, occurring in approximately 25-40% of stroke survivors [24] and it can negatively affect patients' functional recovery and quality of life [25]. The IHMD uses the Patient Health Questionnaire (PHQ-9) [26] to screen for depression at baseline (Week 1-2) and 3-months. The PHQ-9 has been validated for telephonic administration [27] and its test-retest reliability [28] and predictive validity [29] are well characterized.

The IHMD screens for self-care/disability as recurrent disability is common after acute medical illness and hospitalization [30,31]. Moreover, frequent assessment of physical function is consistent with clinical practice in which therapists typically establish functional goals and monitor patient progress toward those goals. The IHMD automates some of this monitoring for the teletherapist. To assess the self-care/mobility, we use questions on lower extremity strength, self-care tasks, and mobility from the 16-item version of the Stroke Impact Scale (SIS-16) [32]. The SIS-16 is a psychometrically robust stroke-specific outcome measure and it has been validated for telephonic administration and its test-retest reliability is good [32]. Different questions are administered on different days to minimize participant burden, but all items are administered approximately every 2 weeks. If the participant screens positive, additional questions are used to determine whether this is a change over the preceding 2 weeks, the extent of the change, and if this is a change for the better or worse.

Once weekly, the IHMD screens for falls. Stroke patients have a four-fold increase in falls risk [33]. One study found that 73% of community dwelling stroke survivors reported falls [34]. Self-report of falls and near-falls on a daily or weekly basis has a long tradition of use in falls research [35]. The falls dialogue has three questions derived from previous research to determine if the participant has recently fallen or had a near fall, the number of times they might have fallen, and the scenario when they fell or almost fell (e.g., "unsteady when moving from bed to chair"; "bumped into things") [36]. Depending on the response, the teletherapist determines if the participant needs to be contacted prior to the next televisit and if the physician needs to be notified.

In addition to screening for depression, self-care/disability, and falls, the IHMD monitors exercise adherence, with the added advantage of providing instant feedback to the participant. For those reporting exercises at or near goal, the feedback is in the form of positive feedback and encouragement such as "You are doing a great job!". For participants recording exercises below goal, the IHMD ascertains the presence of potential barriers such as illness, pain, lack of energy or motivation. Appropriate messages that convey empathy with encouragement are delivered. A default option for report of not performing exercises is receipt of a personalized telephone call ("We are sorry you are having these difficulties, would you like the teletherapist to call you so that we can talk about them and try to get you back on track?").

#### Primary and Secondary Outcome Measures

Self-report instruments (surveys) are used to evaluate the primary and secondary outcome measures at baseline, 3-months and 6-months. Participants are administered the self-report instruments through telephone interviews executed by a research assistant located at the Coordinating Center, who is blinded to the study group assignment. Table 3 presents detailed descriptions of the self-report instruments used to answer the primary and secondary research questions. A brief description of each instrument follows. The motor subscale of the FONEFIM [6,7] is the primary outcome measure. There are four different instruments used to assess each of the four secondary research questions. The Late-Life Function and Disability Instrument [8] is a two-part instrument used to assess disability (altered performance of major life tasks and social roles) and functional limitations (altered ability to perform specific activities encountered in daily actions) [8,37]. The Falls Self Efficacy Scale [9] is used to measure the fear of falling. Reker et al.'s Stroke-Specific Patient Satisfaction with Care is used to measure the participants' satisfaction [10].

**Table 3:** Study instruments used to measure the outcomes

1.	FONEFIM Motor subscale of the Telephone Version of the Functional Independence Measure
	The FONEFIM was developed as a telephonic alternative to the in-person, performance based Functional Independence Measure (FIM). It shows good concordance with the in-person FIM [6,7]. The FONEFIM instrument includes 18-items, each with a maximum rating of 7 (complete independence) and a minimum rating of 1 (complete dependence). Thirteen of the 18-items assess motor functioning encompassing four categories (FIM-motor): 1) self-care (e.g., bathing); 2) sphincter control (e.g., bladder management); 3) mobility (transfers to bed/chair/wheelchair); and 4) locomotion (e.g., walking). The motor subscale scores range from 13 to 91.

2.	**LLFDI** Late Life Function and Disability Instrument

	The LLFDI was developed as a comprehensive assessment of function and disability in community-dwelling older adults [8]. The disability component assesses self-reported limitation (capability) and frequency (performance) of engaging in 16 major life tasks. The functional construct evaluates self-reported difficulty in performing 32 physical activities, comprising three domains: upper extremity, basic lower extremity, and advanced lower extremity. The raw scores from each item response are transformed into linear scaled scores (0 to 100) and accordingly summed to represent component and domain values.

3.	**FES** Falls Self Efficacy Scales

	The FES is a 10-item scale that assesses the impact of fear of falling on a person's confidence to perform everyday tasks. An example of one of the items is: "How confident are you that you can take a bath or shower without falling?" Study participants rate each question on a scale of 0 to 10, and the scores are aggregated to give a total score between 0 (low fall-related self-efficacy) and 100 (high fall-related self-efficacy, that is very confident of not falling). The FES has good internal consistency (α = .91), test-retest reliability (r = .71), and construct validity [9].

4.	**SSPSC** Stroke Specific Patient Satisfaction with Care

	The SSPSC uses 15 items to assess three dimensions of satisfaction (nine items on hospital care, four items on home-based care, and two items on overall care) [10]. Each of the 15 items is scored using a Likert scale ranging from 1-4.

#### Control Variables

Sociodemographic variables (age, sex, race, education, income, marital status, and number of people living with the participant) are collected via self-report. Several clinical variables are collected at baseline as well. Stroke severity is measured by the Goldstein and Chilukuri scale, which is an algorithm to derive the Canadian Neurological Scale (CNS) retrospectively based on information in the patient's medical record [38]. The CNS, a highly reliable and valid stroke scoring system [39], focuses on level of consciousness, speech and strength. The aggregate physical dimension score from the Stroke Impact Scale (SIS) 2.0 is used at baseline to assess stroke related physical impairment [40]. The physical dimension score is a composite of four primary SIS domains: strength, hand function, ADL/IADL and mobility. The SIS uses a 5-point Likert scale ranging from "not difficult at all" to "cannot do at all." The Geriatric Depression Scale (GDS) is employed to measure depression [41]. The GDS is a 30-item scale, where each item is measured on a "yes/no" format. Scores range from 0 to 30, with higher values reflecting greater levels of depression.

#### Diary

Participants in both the TR and Usual Care groups track receipt of therapy services outside of the study intervention via a weekly diary for the entire three months of the intervention period. In this weekly diary, participants record receipt of physical or occupational therapy (yes or no). If yes, the participants are to include the time (in minutes) spent in receipt of therapy services, and the location of that therapy (VA hospital, VA outpatient facility, non-VA facility, and home). Participants are also asked if they have fallen or nearly fallen in their home, and the number of falls they experienced. The diary records whether or not they performed any study exercises (as prescribed by the teletherapist), or other exercises (those assigned outside of the study protocol), the number of minutes and number of times per day that they did so.

#### Exit Interview

Participants in the TR group are administered an exit interview after the 3-month outcome measure instruments have been completed. This interview is performed via telephone by the Coordinating Center. The interview includes 13 closed-ended questions using a 5-point Likert-type scale with different response options (e.g., "very unsatisfied" to "very satisfied"). The closed-ended questions assess the participant's view toward the **general intervention **(e.g., "How would you rate your overall satisfaction with the in-home intervention?"), **equipment **(e.g., "How comfortable were you being videotaped and then talking with the therapist?"), specific components of the **physical function component of the intervention **(e.g., "How useful was the toilet and tub/shower training for you?"), and **exercise component of the intervention **(e.g., "How often do you think you will apply what you learned from the exercise training in the future?"). In addition, there are four-open ended questions (e.g., "If you could change one thing about the whole intervention, what would it be?").

### Sample Size Determination and Statistical Power

The primary research question is: Over the study period, does the TR group have greater improvement in function (motor sub-scale of the FONEFIM) than the Usual Care group?

In our sample size calculations, we assume a standardized difference of 0.50 in the motor FIM score from baseline to 6 months, based on a randomized trial of a combination of assistive technology and exercise intervention similar to our study, that used a measure of ADL similar to the motor FIM over 6 months [42]. In a sample of 163 patients with a first ever stroke admitted for inpatient rehabilitation, Schepers et al. [42] reported a standard deviation of 8.3 for the unadjusted FIM motor domain for stroke patients. Based on a standard deviation adjusted to account for correlation between repeated measurements of the outcome, and using an independent samples t-test with a 0.05 two-sided significance level and 80% power, a sample size of 20 patients per group is required to detect a mean difference of 9.2 or higher in the FIM motor score between the intervention and control groups. Because we assume an approximate dropout rate of 20%, we are recruiting approximately 25 stroke patients per arm. We will test for baseline imbalances. If we find significant differences between the groups on baseline factors, we will control for these in secondary analyses to determine if these may account for between group differences in study outcomes.

If the proportion of drop outs are missing at random or missing completely at random, the likelihood-based mixed model for repeated measures will be applied for the data analysis. Multiple imputation techniques will also be applied to replace the missing data for both the cases that are considered to be drop outs and deaths. The results of the analyses using both the imputed and un-imputed data will be compared. If were to suspect that these analytical techniques were to yield missing not at random (MNAR) drop-out mechanisms or deaths, then we will try sensitivity analysis approaches based on the best scenario or worst scenario assumptions.

### Primary Analyses

The unit of analysis is the participant. Because the motor subscale of the FONEFIM, a continuous variable, is measured at baseline, 3-, and 6-months, we will use a linear mixed-effects model. Fixed effects in the model will include treatment (TR or Usual Care), RBU, (with or without), time (3-and 6 -months), and the interactions between treatment and RBU and between treatment and time. Participant level random effects (e.g., patient age; stroke severity; elapsed time between stroke onset and enrollment in the study) will be included in the model to account for correlations between participants' repeated measures over time. Primary analyses will be conducted as intent-to-treat, and sensitivity analyses will examine the implications of the intent-to-treat assumption.

### Secondary Analyses

The secondary research questions are whether or not, over the 6-month study period, the participants who received the TR intervention have greater improvements in disability (disability component of the Late Life Functional Disability Instrument), falls related self-efficacy (Falls Self Efficacy Scale), and patient satisfaction (Stroke Specific Patient Satisfaction with Care Scale), compared to participants in the Usual Care group. Since these secondary outcomes are continuous and are measured at three time points for each participant, we will use linear mixed-effects models. A final secondary research question is whether the participants discharged from a VAMC with a RBU have greater improvements in function (physical function of the motor sub-scale of the FONEFIM) than participants discharged from a VAMC without a RBU. This latter question will be addressed in the primary analysis in which we will consider the interaction between treatment (TR intervention group or Usual Care group) and RBU (with or without).

None of the four Institutional Review Boards (at the Coordinating Center and the three individual study sites) that we obtained approval from nor the funding agency believed that a DSMB was required. Despite the fact that there was a decision to not have a formal DSMB at the outset of the trial, we did decide a priori to have an independent review of the safety of the participants during the trial. The PI of the study will do a mid-point review with regards to the prevalence of potential harms (hospitalizations, falls, stroke, death). At this mid-point review, the PI will conceal the codes from the rest of the study team of identification of participants. We track adverse events and protocol violations per standard IRB requirements. Adverse events, protocol violations, and anything out of the ordinary related to the protocol are reviewed in bi-weekly meetings with the PIs. Should there be any evidence of unusual adverse events, we will engage an external group to review these.

All Adverse Events regardless of seriousness or relationship to study procedures are entered on the database. The adverse event entered specifies the date of onset, action taken with respect to study procedures, corrective treatment/therapy given, outcome and his/her opinion as to whether the Adverse Event related to the intervention. The study coordinator at the Coordinating Center keeps a log of all adverse events, the date the event occurred and the date of the learned event. This log also differentiates such events by whether they are considered to be serious adverse events and whether or not they are related to the intervention. The study coordinator, in turn, reports the adverse events to the local institutional review board. The study coordinator also documents the participant withdrawals and determines and documents whether it is due to the intervention.

## Discussion

TR has the potential to be integrated into mainstream allied health practice with improvements in efficiency and effectiveness of care [43]. However, TR interventions must be examined critically using rigorous scientific methodology. A 2009 published systematic review of clinical outcomes, clinical process, and healthcare utilization and costs associated with TR included 28 articles, but only 8 included RCTs. Of the 8 RCTs, none reported on individuals post-stroke [44]. This study addresses a significant knowledge gap by applying rigorous clinical trial methodology to determine the effect of a telehealth intervention on rehabilitation outcomes for post-stroke patients after discharge to home.

The use of TR to address pressing patient care needs has been identified as one of five major priority areas of future development by the Department of Veterans Affairs [45]. Even though there is a burgeoning use of TR to employ clinical services within and outside of the VA for such populations as spinal cord injury and wound care [46,47], future program developers could use our findings to implement more effective and efficient strategies for rehabilitation service delivery in the home.

## Conclusions

The results of the study may support the creation of national partnerships to implement more efficient and effective approaches to coordination of care when transitioning patients post-stroke from hospital to home. Findings from the study have potential applicability to other rehabilitation patient populations (e.g., spinal cord injury) and possible utility for community dwelling individuals with mobility disability. If the TR intervention proves effective, it could help reduce future healthcare costs associated with the long-term effects of stroke patients living in the community with residual disability.

## Competing interests

The authors declare that they have no competing interests.

## Authors' contributions

NC, DR, PG, PQ, MM, JS and HH contributed to the design of the study. All authors contributed to the creation of the Manual of Procedures, implementation of the study protocol and acquisition of data. NC and HH drafted the manuscript, and all authors provided critical revision and have approved the final manuscript.
